# Mini-Plays: An Innovative Method to Improve Clinician Confidence in Advance Directive Discussions

**DOI:** 10.1093/geroni/igaf122.1813

**Published:** 2025-12-31

**Authors:** Sara Bybee, Jacqueline Telonidis, Jacqueline Eaton, Nicole Fleming, Katherine Supiano

**Affiliations:** University of Utah, Salt Lake City, Utah, United States; University of Utah, Salt Lake City, Utah, United States; University of Utah, Salt Lake City, Utah, United States; University of Utah, Salt Lake City, Utah, United States; University of Utah, Salt Lake City, Utah, United States

## Abstract

For health care clinicians, there are few training opportunities on end-of-life or advance directive conversations with patients and families, especially with lesbian, gay, bisexual, transgender, queer, and other sexual and gender minority (LGBTQ+) persons. To address these gaps, the Hospice Foundation of America’s Advance Directive Mini-Plays were produced during a hybrid continuing education event. Mini-plays (“Lily” and “Room 402”) written by playwright Bryan Harnetiaux, were performed by two actors during a continuing education event for clinicians working in hospice-related contexts. Afterwards, a brief discussion was held, and attendees completed a REDCap questionnaire regarding their satisfaction and confidence level using a 5-point scale (1=not at all true and 5=completely true). Fifty attendees completed post-surveys. The majority were social workers (62.0%), followed by other mental health clinicians (14.0%), other health care clinicians (10.0%), chaplains (6.0%) and nurses (2.0%). Nearly all (96%) reported they would recommend this event to colleagues. Clinicians (84%) reported “very true” or “completely true” in response to the statement, “The mini-plays were an engaging way to learn about advance directives,” and 80% reported “very true” or “completely true” in response to the statement, “The mini-plays improved my understanding of advance care planning.” Implications include that advance directive mini-plays may be effective in conveying the importance of advance care planning and in training clinicians to foster these discussions with patients and their chosen support persons. Open-ended responses illuminated the difficulties in providing this content electronically, which may suggest the need for future productions of the mini-plays to be performed in-person.

